# Psychometric characteristics of the Mini-TEA scale: a screening instrument for autism spectrum disorder in children

**DOI:** 10.1016/j.jped.2025.05.006

**Published:** 2025-06-26

**Authors:** Cassiano Mateus Forcelini, Regina Ampese, Helena Younes de Melo, Camila Pereira Neubauer Pasin, José Renato Donadussi Pádua, Itamara Danelli de Moura, Camila Boschetti Spanholo, Francine Ehrhardt Hoffmann, Júlia Breitenbach Diniz, Laís Cristine Zanella Capponi, Luiza Souza, Maxciel Zortea

**Affiliations:** a Associação de Pais e Amigos dos Excepcionais (APAE), Passo Fundo, RS, Brazil; b Universidade de Passo Fundo, Escola de Medicina, Passo Fundo, RS, Brazil; c Universidade do Valo do Rio dos Sinos, São Leopoldo RS, Brazil

**Keywords:** Autism, Screening, Sensitivity, Specificity, Validation, Mini-TEA scale

## Abstract

**Objective:**

Early diagnosis of autism spectrum disorder (ASD) is advisable to promote better prognosis. The Mini-TEA scale was conceived as a sensitive screening for ASD among children. The authors aimed to confirm the diagnostic accuracy of the scale in a wider population.

**Method:**

279 children from 2.5 to 12 yo were recruited, most of them under evaluation for possible ASD in the APAE of Passo Fundo/RS, as well as children with other diagnoses and normal children. Their parents/relatives answered the 48 binary questions (yes/no) of the Mini-TEA scale, divided into 15 items, which resulted in a score from 0 to 15. After that, the children were evaluated regarding the diagnostic criteria of ASD by experienced raters (gold standard) who had previously submitted to a concordance test and remained unaware of the children’s scores. Sensitivity and specificity Figs. were obtained. Factor analysis and Item Response Theory approaches were used for validity evidence.

**Results:**

115 children were diagnosed with ASD. Scores ≥9 had 98.3 % of sensitivity and 62.2 % of specificity for the diagnosis. Two cases with the typical presentation of Asperger’s syndrome scored lower than 9. The mean time for screening was about 8.5 min. The validation model presented excellent coefficients of factorability. The analysis showed that the total variance of the scores of the scale through the 15 items was explained only by the set of ASD symptoms (unidimensionality).

**Conclusion:**

The Mini-TEA scale is a very sensitive tool to screen for ASD and has high internal consistency for assessing typical autistic symptoms.

## Introduction

Autism Spectrum Disorder (ASD) is characterized by persistent deficits in social communication and interaction across multiple contexts, as well as restricted, repetitive patterns of behavior, interests, or activities, all these symptoms causing impairment in social, occupational, or other important areas of current functioning.^1^ Early diagnosis of ASD is advisable because a better prognosis of development emanates from several evidence-based interventions that should begin as soon as possible.^2^^,^^3^ However, there is a scarcity of services with trained professionals for the adequate diagnosis of ASD in Brazil,^4^ as well as in other developing countries.^5^

The employment of screening tools could be an aid to separate those infants and children who actually need further evaluation from those whose suspicion of ASD is not appropriate, especially in the context of limited health resources. In this setting, the widespread use of the revised form of the Modified Checklist for Autism (M-CHAT-R/F) in toddlers has been a recommendation for pediatricians.^6^^,^^7^ However, this instrument is devoted only to toddlers from 16 to 30 months of age, a population that often skips such evaluation due to a lack of screening through adequate pediatric care.^8^

This scenario led the group to develop the Mini-TEA scale, a screening tool for ASD in Brazilian Portuguese directed to parents/relatives of children from 2.5 to 12 yo, because of the lack of such a scale for this age group in Brazilian Portuguese, Initial results of the present research suggested that the scale has an excellent sensitivity (100 %) and a reasonable specificity (68 %),^9^ enabling the proposal of its use among children not previously assessed with the M-CHAT-R/F. This research has been extended to embrace a wider population in order to confirm the diagnostic accuracy and to ascertain other characteristics related to the validation process of the Mini-TEA scale. The purpose of this manuscript is to report the results of this survey extension and reinforce the helpfulness of the Mini-TEA scale to help in the screening for ASD among children.

## Methods

The local ethics committee approved this cross-sectional study for evaluating the accuracy of the Mini-TEA scale in July 2023 (approval number 6.175.425). The study was accomplished from July 2023 to July 2024 in the *Associação de Pais e Amigos dos Excepcionais* (APAE - Passo Fundo, RS, Brazil). The APAE from Passo Fundo houses a *Centro Regional de Referência em Transtorno do Espectro Autista* (Regional Reference Center for ASD) of the *Programa TEAcolhe,* a program for improving diagnosis and management of ASD supported by the Government of Rio Grande do Sul (RS), Brazil.

Children (and their parents/relatives) who were under evaluation for possible ASD and other neurodevelopmental disorders in the APAE were recruited upon invitation. In parallel, other parents/relatives brought normal children on their own initiative attracted by local advertisements. This convenience sample included children aged from 2.5 to 12 yo. The written consent was obtained from the child’s legal guardians and, whenever feasible, from the child. One child declined participation. The only exclusion criterion was guardians’ illiteracy.

The first step was the obtainment of demographic and clinical data from an interview with each child’s parents/relatives. After that, they were asked by medical students about the 48 binary questions (“yes” or “no”) of the Mini-TEA scale, divided into 15 items.^9^ Finally, the child was evaluated by a rater (a pediatric neurologist or a psychologist, both experienced in TEA) regarding the diagnostic criteria of ASD from the DSM-V-RV.^1^ The raters remained unaware of the children’s scores on the Mini-TEA scale until the end of the study. They were previously submitted to a concordance test (kappa statistics) to ascertain that they scored similarly 20 children in relation to the clinical diagnosis of ASD.

The authors estimated the sample size based on the study of Kyriazos for a factorial exploratory analysis of binary data,^10^ which suggested at least 200 participants, and the study of Sahin & Anil that considered an adequate sample size of 250 participants for the application of Item Response Theory over unidimensional instruments.^11^ The final sample size was estimated to be circa 280 participants, adding 12 % to prevent losses due to missing data.

Ultimately, the results were assessed to define the primary outcome: the cut-off score that could offer a high sensitivity for screening to ASD and an acceptable specificity considering the diagnostic criteria according to DSM-V-RV as the gold standard. To examine the Mini-TEA scale as a test-criteria analysis with an external measure (gold standard), the authors built a receiver operating characteristic (ROC) curve to verify levels of specificity and sensitivity of the scores in identifying cases of ASD established with the DSM-V-TR. Likewise, this analysis allowed an estimate of the instrument cut-off point. The interview duration for applying the mini-TEA scale was recorded as a secondary outcome to offer an estimate of the time spent screening for ASD.

Mean, standard deviation and frequency were used for descriptive purposes of clinical data, according to the nature of the variable. Shapiro-Wilk normality tests and visual inspection of histograms were used to evaluate the distribution of the quantitative variables.

For validity evidence of the Mini-TEA scale, the authors used factor analysis and item response theory (IRT) approaches. First, the dimensionality of the scale was tested with exploratory factor analysis (EFA) with unweighted least squares (ULS) as the extraction method. Tetrachoric correlations for dichotomous data and parallel analysis as a method to define the number of factors were used.^12^ In addition, the 15 items of the Mini-TEA scale were submitted to an analysis using the Rasch model for dichotomous unidimensional instruments. Infit, outfit (raw and standardized), and signed chi-squared test (Sχ²) were used as fitting coefficients. Values from 0.7 to 1.3 are considered adequate (rule of thumb) for the infit and outfit indices.^13^ The significance level of α = 0.05 and power of 0.8 were used. The statistical programs employed were R Studio (RStudio Team, 2020), using the packages psych (for EFA) and mirt (for Rasch analysis) and Microsoft Excel®.

## Results

The sample comprised 279 children whose parents/relatives answered the Mini-TEA scale. All participants completed the study and were assessed by one of the raters regarding the diagnosis of ASD. The result of the concordance test evidenced an extremely similar evaluation between raters, with Kappa coefficient = 1.0 (*z* = 4.47; *p* < 0.001), denoting that both agreed highly for the diagnosis of ASD.

Table 1 presents detailed information about the 279 participants. 115 had the diagnosis of ASD confirmed. Regarding the severity of ASD, 74 were classified as level 1 (“requiring support”), 30 as level 2 (“requiring substantial support”), and 11 as level 3 (“requiring very substantial support”). Most of them are currently under etiologic investigation. The main alternative diagnoses of ASD were the following: intellectual disabilities, communication disorders, attention-deficit/hyperactivity disorder, and oppositional defiant disorder. These children were referred to medical accompaniment. Learning, behavior, and speech problems were the leading symptoms that motivated the parents/relatives to seek aid, both from the ASD group and the group with other diagnoses. There were also volunteers without any complaints who contributed to the study sample.

**Table 1:** Clinical characteristics of the sample.

	ASD children (*n* = 115)				Non-ASD children (*n* = 164)			
Age (years)	6.18 ± 2.79				6.75 ± 3.07			
Mini-TEA score	10.98 ± 4.54				9.84 ± 5.02			
Sex	Male		Female		Male		Female	
	98		17		110		54	
				
	Yes		No		Yes		No	
Learning problems	94		21		77		87	
Behavior problems	113		2		97		67	
Speaking problems	109		6		53		111	

					Children with other diagnoses (*n* = 100)		Normal children (*n* = 64)	
Age (years)					5.97 ± 2.78		7.26 ± 3.18	
Mini-TEA score					10.24 ± 3.83		4.12 ± 3.40	
Sex					Male	Female	Male	Female
					70	30	42	22

Continuous variables are expressed in mean ± standard deviation, while categorical data are described with absolute number.

Note: ASD, autism spectrum disorder.

**Figure 1:**
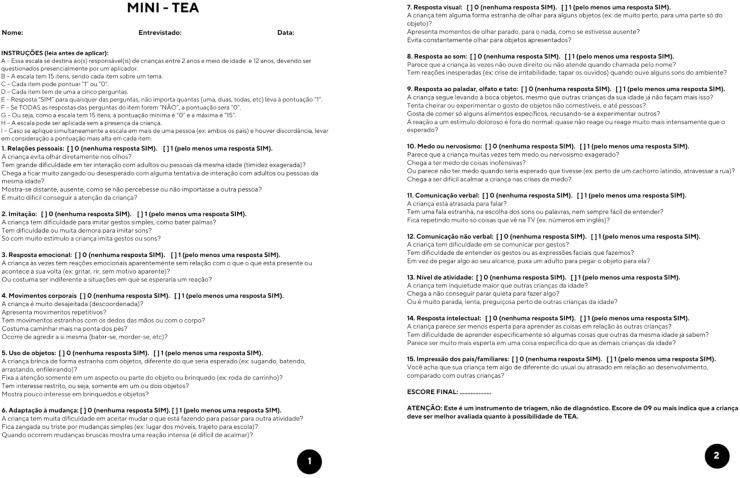
The mini-TEA scale.

The Mini-TEA scale, with 48 questions distributed along the 15 items (Figure 1), was performed with a mean time of about 8.5 min.

**Figure 2:**
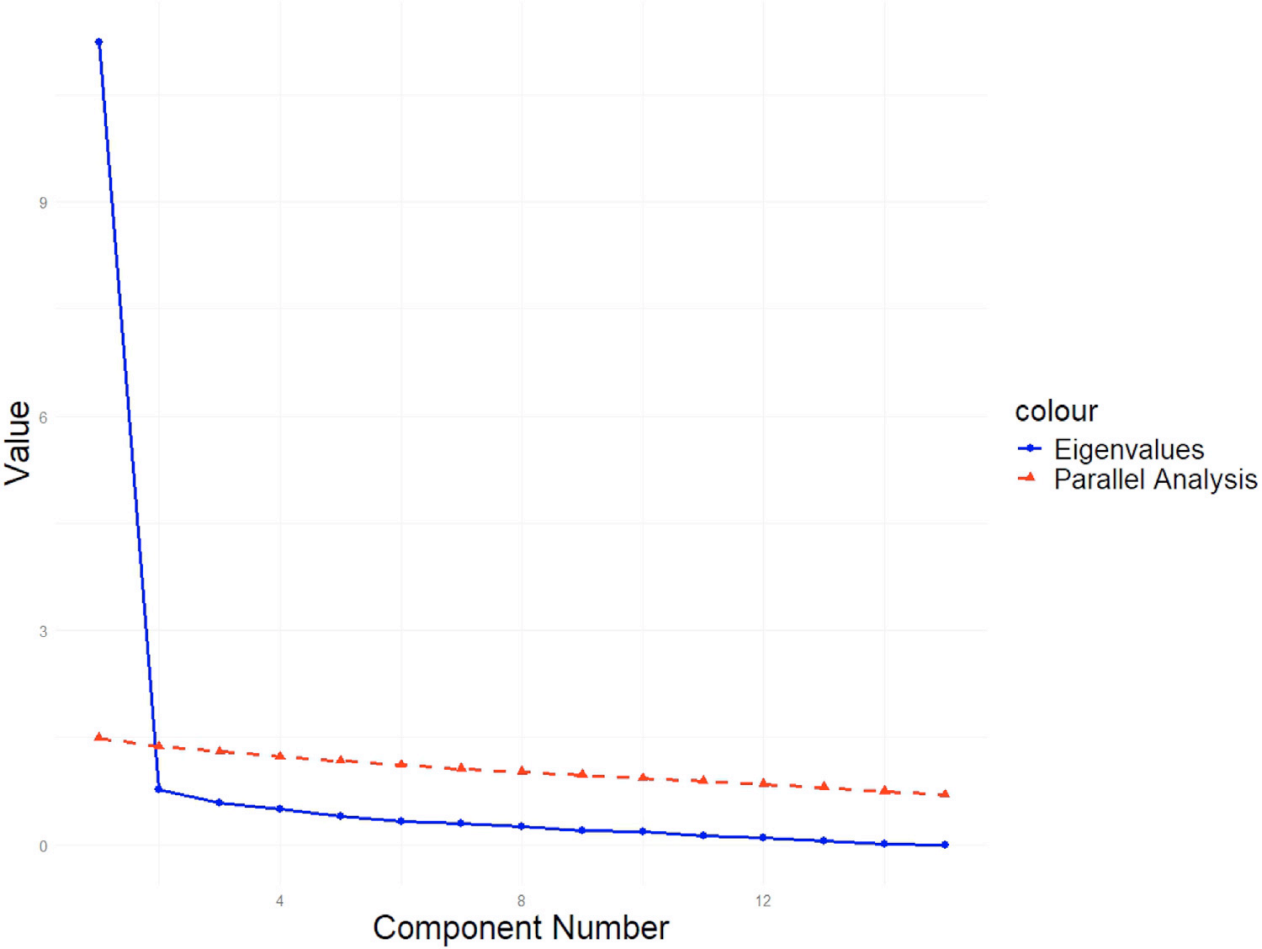
Screen plot for the Mini-TEA scale presenting eingenvalues (in blue) and parallel analysis (in red). The graph reveals that 1 component adds enough information for the model, reinforcing the unidimensionality of the scale.

The EFA model was applied to the scale and presented excellent coefficients of factorability (Bartlett's K² = 38.37 [df = 14; *p* < 0.001] and Kaiser-Meyer-Olkin [KMO] = 0.94). The analysis based on the tetrachoric correlations matrix showed that the total variance of the scores of the Mini-TEA scale through the 15 items was explained by only one factor, that is, the set of ASD symptoms, attesting to the unidimensionality of the instrument. Figure 2 shows the screen plot with eigenvalues and the parallel analysis, revealing that one-factor solution is the best for the present data. The variance explained was 0.73 (ss loadings = 10.98). Table 2 illustrates the loadings of each item for this factor, which are high overall. This denotes that all items reflect the core symptoms of ASD and contribute similarly to a singular score.

**Table 2:** Loadings for the unifactorial solution of the Mini-TEA scale.

Item	Loadings
Item 1	0.891
Item 2	0.881
Item 3	0.952
Item 4	0.911
Item 5	0.906
Item 6	0.823
Item 7	0.888
Item 8	0.778
Item 9	0.815
Item 10	0.865
Item 11	0.810
Item 12	0.839
Item 13	0.831
Item 14	0.768
Item 15	0.856

Note: Loadings values range from 0 to 1.

Considering the unidimensionality of the Mini-TEA scale, the authors implemented the Rasch model to investigate the item’s difficulty (*b*), that is, the percent subjects that answered the item correctly (the percentage that was attributed the ASD characteristic measured by the item) by the percent of subjects that answered incorrect (did not endorsed the item). B-values for the 15 items ranged from −0.706 to 2.796 (mean = 1.61; SD = 0.94). In order to evaluate how well each item fitted accurately the unidimensional model, the authors analyzed infit (how close observed values are from expected values, considering the item’s difficulty – how probable it is to be endorsed - and person’s ability – or the level of symptoms of ASD) and outfit (a similar measure, although not considering item’s difficulty and person’s ability – a measure of unexpected errors). As observed, the respondents’ performance for Item 3 did not completely fit the expected Rasch model when infit is considered. However, when outfit is considered, items 1, 2, 3, 4, 6, 7, 9, 10, and 15 had lower values of outfit, which means they overfit the model and may be redundant or too predictable. Nevertheless, considering the z-outfit, only items 2 and 3 can be really considered problematic (lower than 2 SD), which means they present an overfit to the model and do not add much information for the test. When one considers chi-square tests, items 4 and 14 seem to have significantly higher residual values. After checking the item information curves, the analysis revealed the peak of each item curve ranged from −0.7 to 2.8, with a mean peak of 1.61 (SD = 0.95). Overall, these results indicate the Mini-TEA scale provides more information when a person's ability (in this case, symptoms of ASD) is higher [a test information curve of 0 (zero) would be considered the test is maximally informative on a medium level of ASD]. In other words, if items are endorsed it may indicate a high probability of being diagnosed with ASD.

**Figure 3:**
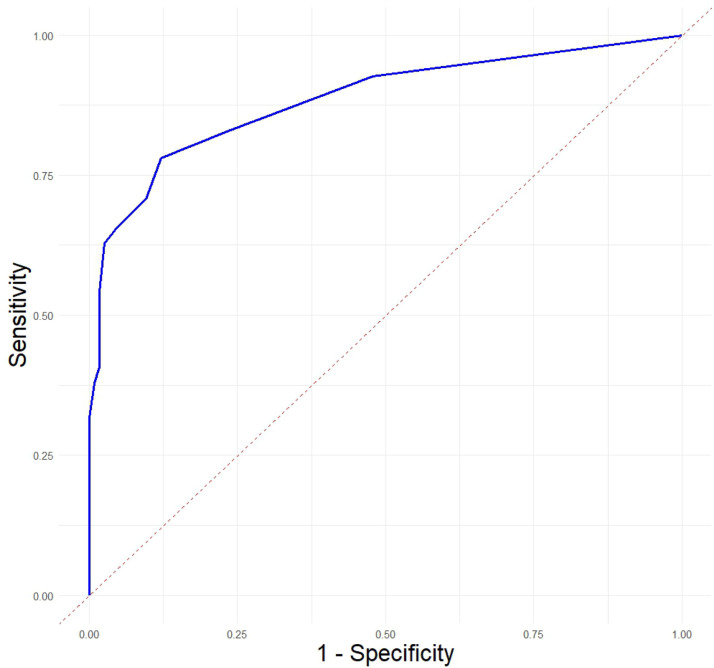
Receiver operating characteristic (ROC) curve for the Mini-TEA scale.

Table 3 presents the sensitivity and specificity of the Mini-TEA scale to predict cases and non-cases of ASD. As a screening test, when sensitivity was prioritized, the cut-off point to identify suspected ASD was proposed: scores equal to 9 or higher had 98.3 % sensitivity and 62.2 % specificity for the diagnosis. Figure 3 illustrates this through the ROC curve, whereas the area under the curve (AUC) value was 0.88, indicating a good discriminating quality. From the 115 ASD children, 58 were scored 15 in the scale, 29 were scored 14, 13 were scored 13, three were scored 12, seven were scored 11, two were scored 10 and on child was scored 9. The only two cases of ASD that were scored <9 on the Mini-TEA scale (two boys, one with “5” and the other with “6”) have the typical presentation of Asperger’s syndrome. On the other hand, normal children were scored from 0 to 11, while those with diagnoses other than that of ASD were scored from 2 to 15.

**Table 3:** Sensitivity and specificity of the Mini-TEA scale (*n* = 279).

Cut-off	Sensitivity	Specificity
15	0.522	1
14	0.765	0.957
13	0.878	0.872
12	0.904	0.817
11	0.957	0.732
10	0.974	0.683
9	0.983	0.622
8	0.983	0.591
7	0.983	0.512
6	0.991	0.457
5	1	0.372
4	1	0.348
3	1	0.293
2	1	0.22
1	1	0.171
0	1	0.073

## Discussion

Improvement in the early diagnosis of ASD is an aimed goal that led to recent worldwide research. Arun and Chavan developed a 37-item questionnaire in India, where the late diagnosis is the rule, with dichotomous yes/no responses to screen for ASD among children aged from 1.5 to 10 years.^5^ They found the Figure of 89 % of sensitivity and specificity. In a different context, a group of researchers created a tool to overcome the delay of more than one year for diagnosis of ASD in the US.^14^ They developed a device to measure eye-tracking-based social visual engagement and found the sensitivity of 70.7 % and specificity of 85.4 % for ASD among 16 to 30-month-old toddlers. Another example is the recently published validation of the Social Communication Questionnaire (SCQ) for the Portuguese population between 4 and 17 y.o.^15^ with values of sensitivity and specificity of 76 % and 93 %, respectively.

In Brazil, despite the identification of ASD before 4 years of age has improved, it still represents only 30 % of the diagnoses made.^16^ In this setting, the authors created the Mini-TEA scale to fill the gap in screening for ASD from 2.5 to 12 y.o. The results here presented confirmed the excellent sensitivity suggested by the previous publication.^9^

The M-CHAT-R/F has been recommended as mandatory to pediatricians for ASD screening,^6^^,^^7^ but its employment is actually far from the recommended widespread use due to a series of reasons: lack of access to adequate pediatric care; medical visits only when the child is ill; short period of consultation in many contexts. As a result, most children in Brazil were not screened for ASD at the time they could be with the M-CHAT-R/F and became older than the age range for which that questionnaire was delineated. The Mini-TEA scale was conceived as a simple tool that can be applied to these children’s parents or relatives not only by medical doctors but also by other health professionals and even by teachers and social agents. This can spread the screening of ASD. On the other hand, almost 60 % of the children without ASD were adequately excluded by the Mini-TEA scale because they scored less than “9”. This is particularly important to decrease the waiting lists of children with suspicion of ASD, especially in the setting of scarcity of trained professionals for adequate diagnosis. All this with a simple and comprehensible questionnaire with dichotomous yes/no responses that take <10 min to be answered by the parents/relatives of children, without the need for the latter’s presence.

Two boys with the typical Asperger’s syndrome skipped the identification among 115 children with ASD. Apart from them, only one child with ASD pointed “9” among the remaining 113. Nevertheless, the authors adjudicate to lower the cut-off point from the previously proposed “10”,^9^ with 97.4 % of sensitivity, to “9” in order to enhance the sensitivity to 98.4 % without prejudice to the reasonable figs. of specificity. For screening purposes, sensitivity should be prioritized.

The failure of the Mini-TEA scale to detect two cases of ASD with a characteristic presentation of Asperger’s syndrome is expected. People with this form of ASD show no language problems and their cognitive development is not marked by an overall delay but by specific impairments in certain areas such as the executive functions, with heterogeneous clinical presentations varying according to age.^17^ Suspicion and screening of Asperger’s syndrome is not easy because of such diversity of clinical manifestations. Consequently, Asperger’s syndrome is often diagnosed belatedly, at 11 years of age on average and even in adulthood in some cases.^17^ There were other children with Asperger’s syndrome in this series, but the possibility of skipping the identification of mild cases of this type of ASD presentation must be kept in mind when employing the Mini-TEA scale for screening purposes. This represents a limitation of the instrument.

The present survey has some limitations to be addressed. The convenience nature of the sample may include selection bias whose impact was partly minimized by a wide number of participants. The authors did not perform an etiologic investigation of the ASD children, but this study was conceived to evaluate the accuracy of the Mini-TEA scale and not to search for causative factors.

The Mini-TEA scale was not the first Brazilian attempt to a screening tool for ASD. In 2008, Sato and cols. published a preliminary study of translation and validation of the Autism Screening Questionnaire (ASQ).^18^ The original research that gave rise to the ASQ was undertaken in 1999 in London.^19^ Both studies were performed in samples derived from a series of pediatric patients with known neuropsychiatric disturbances in order to separate ASD from other diagnoses. That is, ASQ was not designed to be a screening in the general population. Besides, the Brazilian version was tested in children with a restricted mean age of about 10 to 11 yo.

Although ASD is universal, the behavioral manifestation of autistic symptoms may vary according to different cultural contexts.^20^ Taking this into account, a Brazilian group undertook a survey that consisted of the translation of the Childhood Autism Spectrum Test (CAST) to Brazilian Portuguese to investigate the factor structure of parent-reported autistic symptoms in a large sample of children/adolescents from the metropolitan area of São Paulo.,^21^ This provided evidence of the cross-cultural validity of the classical autistic symptoms in the Brazilian urban population.

The authors demonstrated that the total variance of the scores of the Mini-TEA scale was explained by only one factor, that is, the set of ASD symptoms. This was in line with other studies with similar scales and unidimensional factor solutions,^22^ although a number of factors for ASD screening scales are particularly variable in the literature.^23^ Considering this unidimensionality of the Mini-TEA scale, the Rasch model showed that overall the items are adequate to assess ASD, and are more informative (less uncertainty) when individuals present higher levels of symptoms. This should not be mistaken by the cut-off point proposed, because the Rasch model parameters are independent of the sensitivity/specificity analysis (which considers external criterium, i.e. the clinical diagnosis of ASD). The analysis takes the various presentations of the latent trait (informed by the different items of the scale) and tests how probable this item is to be endorsed in the sample. Despite some items could be adjusted or restructured (one must consider they are endorsed based on at least one affirmative answer to the set of questions of that item, therefore questions could be reanalyzed in future analysis), most of the fit indices indicated items were suitable for the one-factor solution. Besides, based on this analysis, researchers and practitioners could look at each item separately in order to use them as a more precise guide to inform about the diagnosis and clinical impact of decisions and interventions.^24^

In summary, the authors concluded that the Mini-TEA scale is a very sensitive tool to screen for ASD and has high internal consistency. The widespread use of this scale may be helpful for purposes of early identification of suspected cases of ASD and excluding non-ASD cases in several contexts.

## AI declaration

Generative artificial intelligence was not employed to write this manuscript.

## Conflicts of interest

The authors declare no conflicts of interest.
